# Effects of Intrathecal Morphine Administration in Patients Undergoing Primary Total Hip Arthroplasty Under Spinal Anesthesia With Quadratus Lumborum Block for Postoperative Analgesia

**DOI:** 10.7759/cureus.57346

**Published:** 2024-03-31

**Authors:** Promil Kukreja, Kevin O'keefe, Jacelyn E Peabody Lever, Hanna Hussey, Paul D Piennette, Brooke Vining, Peter Nagi, Roland T Short, Scott Mabry, Hari Kalagara

**Affiliations:** 1 Anesthesiology and Perioperative Medicine, University of Alabama at Birmingham (UAB), Birmingham, USA; 2 Anesthesiology and Perioperative Medicine, University of Alabama at Birmingham (UAB) School of Medicine, Birmingham, USA; 3 Orthopaedics, University of Alabama at Birmingham (UAB), Birmingham, USA; 4 Department of Anesthesiology and Perioperative Medicine, Mayo Clinic, Jacksonville, USA

**Keywords:** regional anesthesia, neuraxial anesthesia, quadratus lumborum block, intrathecal morphine, neuraxial opioids, post-operative analgesia, total hip arthroplasty

## Abstract

Introduction

Quadratus lumborum (QL) block has previously been shown to provide improved analgesia in patients undergoing primary total hip arthroplasty (THA) under spinal anesthesia when compared to spinal anesthesia alone. Additionally, recent studies have shown the addition of intrathecal morphine (ITM) to provide superior postoperative analgesia in patients undergoing various surgical interventions including total knee arthroplasty under spinal anesthesia with peripheral nerve blockade. At this time, however, there has not been a study evaluating the effects of intrathecal morphine in patients undergoing THA under spinal anesthesia with QL block. This study aims to assess if the addition of intrathecal morphine can provide adequate or even superior postoperative analgesia in patients undergoing primary THA.

Methods

This retrospective study included 26 patients in the spinal/QL block/intrathecal morphine (SA+QLB+ITM) group, 31 patients in the spinal/QL block group (SA+QLB), and 28 patients in the spinal only (SA or control) group. Twenty-six patients undergoing primary THA under a combination of spinal anesthesia and peripheral nerve blockade (quadratus lumborum block) were given a dose of 100 mcg of intrathecal morphine. Various parameters were evaluated including Post-Anesthesia Care Unit (PACU) and 24-hour visual analog scale (VAS) scores, time to first opioid use, 24- and 48-hour total opioid use as oral morphine equivalents (OME), 24-hour ambulation distance, and time from block placement to hospital discharge. The results were analyzed and compared to patients undergoing primary THA under spinal anesthesia with QL block (no intrathecal morphine) and compared to a control group of patients undergoing primary THA under spinal anesthesia only.

Results

The study analysis included 26 patients in the SA+QLB+ITM group, 31 patients in the SA+QLB group, and 28 patients in the SA (control) group. When compared with the control group, the SA+QLB+ITM had lower 24-hour total opioid usage (mean difference 20.80 OME, CI 6.454 to 35.15, p-value 0.0025), longer time to 1st opioid use (mean difference -20.51 hours later, p-value .0052), lower 24-hr VAS (difference 2.421, p-value 0.0012, CI 0.8559 to 3.987), and faster time to discharge (16.00 hr earlier, p-value 0.0459). When compared to the SA+QLB group, the SA+QLB+ITM group only showed a statistically significant difference in faster time to discharge (19.46 hr earlier, p-value 0.0068). However, while there was no statistically significant difference in time to 1st opioid use between the control and SA+QLB group, the difference did become significant when comparing the control to the SA+QLB+ITM group (mean difference -20.51 hours later (p-value .0052). There was no significant difference in either of the three groups in ambulation distance at 24 hours, PACU VAS, or 48-hour total opioid use.

Conclusion

Our study concludes that the addition of 100 mcg ITM for total hip arthroplasty under spinal anesthesia improved postoperative analgesia compared to the control group. Also, the ITM group did better with respect to delay in first opioid use and decreased hospital stay compared to the control and block-only groups. Our study warrants no more concerns of PONV, pruritus, or respiratory depression with this dose of ITM and requires standard postoperative care.

## Introduction

Osteoarthritis (OA) continues to be an ever-present source of debilitation and diminished quality of life in both the obese and elderly populations. Arthroplasty continues to be a popular and effective treatment for the pain and decreased function associated with OA. Specifically, total hip arthroplasty (THA) continues to be one of the most frequently performed procedures in the United States [1-3]. It is estimated that more than 450,000 of these procedures are performed in the US per year, and this is projected to increase to 635,000 by the year 2030 [4].

Postoperative pain control is paramount in THA as it has been associated with earlier mobilization, decreased length of hospital stay, and increased patient satisfaction [5]. Furthermore, there is a known negative effect of severe postoperative pain on patient outcomes and morbidity, many of which are sequelae of limited mobility and extended hospital stays [6]. While this is known, the management of pain in patients undergoing THA has often been challenging for various reasons due to the complex innervation of the hip joint and surrounding soft tissues. Additionally, there is a need to balance analgesia with preservation (or early return) of motor function in order to facilitate early mobilization.

With the increasing incidence of patient comorbidities, performing THA under various combinations of spinal anesthesia and peripheral nerve blockade has emerged as a viable alternative to general anesthesia, and many regional techniques have been described to assist in postoperative pain control [7]. Quadratus Lumborum (QL) block has emerged as a favorable technique, providing a balance of analgesia and intact motor function. Multiple studies have shown QL block to provide a reduction in pain, opioid consumption, earlier mobilization, and earlier discharge [8-10].

Intrathecal morphine (ITM) has been studied in hip arthroplasty as far back as 1993. Many studies have since demonstrated benefits regarding pain control, but often with side effects of pruritis, postoperative nausea and vomiting (PONV), and respiratory depression [11]. A 2021 meta-analysis by Gonvers et al. showed good evidence that intrathecal morphine provides effective analgesia after lower limb arthroplasty without an increased risk of respiratory depression but with an increased rate of PONV [12]. Additionally, this meta-analysis showed that 100 mcg functions as both a ceiling dose for analgesia and an increased rate of PONV, but more recent studies have indicated 150 mcg to have similar side effect profiles but improved analgesia [12,13]. The mechanism of action of IT opioids is complex.

Recent studies have launched into further evaluation of the potential benefits of combining the techniques of ITM and peripheral nerve blockade in patients undergoing lower limb arthroplasty under spinal anesthesia. In a study examining total knee arthroplasty, the addition of ITM to peripheral nerve blockade and spinal anesthesia improved postoperative analgesia and reduced opioid consumption [14]. No similar study evaluating total hip arthroplasty has been completed. ITM and QL block are separate modalities that both have demonstrated benefits in patients undergoing THA under spinal anesthesia. However, to our knowledge, no study has evaluated the effect of the combination of ITM and QL block. Our aim is to evaluate potential synergistic vs unfavorable effects that the combination may provide to patients undergoing primary THA under spinal anesthesia.

## Materials and methods

In this retrospective study, approved by the Institutional Review Board (IRB) at the University of Alabama at Birmingham (IRB 300000976), we selected three groups of patients who had undergone primary total hip replacement. In this study, we conducted a retrospective chart review of 85 patients undergoing THA. The study was powered based on our previous prospective randomized trial [8]. These THA patients were subdivided into three groups based on pre-operative intervention; the first group (SA) did not receive a QL block nor intrathecal morphine (N=28 control), the second group had a QL block (SA+QLB) only (N=31), and the third group had a QL block and ITM (SA+QLB+ITM) prior to THA (N=26).

The Quadratus Lumborum (QL) block performed in this study is an ultrasound-guided transmuscular block where local anesthetic was injected at the fascial plane between the QL muscle and the psoas major muscle [8]. The QL was performed preoperatively in our regional block area. Patients were consented to the block and the site confirmed during time-out for the block. Both groups with QL block received 50-100 mcg of intravenous fentanyl for sedation during the block. The patient was positioned in the lateral position, and a curvilinear low-frequency probe was used to identify the target. Both groups received a bolus of 25 ml of 0.2% ropivacaine for the QL block. The ITM group received 100 mcg of preservative-free morphine mixed with a spinal dose of local anesthetic. Inclusion criteria consisted of any adult patient 18 years or older, who underwent primary total hip arthroplasty. Rescue pain medication was given orally either as oxycodone or hydrocodone and oral morphine equivalent (OME) was calculated for comparison between the two groups. Exclusion criteria included patients with a past medical history of chronic pain or chronic opioid use greater than 30 days and patients undergoing any surgeries other than primary THA.

Data collection, statistical analysis, and data presentation

Our primary outcome measures were the cumulative oral morphine equivalents (OME) used by patients at 24 and 48 hours and pain score assessed by the visual analog scale (VAS) in the post-anesthesia care unit (PACU) and 24-hour post-operative. The secondary outcome measures were time to first opioid use, distance ambulation at 24 hours post-operative, and length of hospitalization. Authorized personnel conducted a chart review in the electronic medical record (EMR) to extract outcome data for this retrospective study per IRB-approved protocol. Data are presented as the mean and standard error of the mean for continuous variables or the number and percentage of total for categorical variables. Group comparisons were conducted using either ANOVA with Tukey’s multiple comparison test for parametric data or Krustal-Wallis with Dunn’s multiple comparison tests for nonparametric endpoints. A p-value of less than 0.05 was considered statistically significant. All statistical analyses and graph generation were carried out using Graphpad Prism version 9.3.1 for Mac OS X (Graphpad, Boston, MA).

## Results

In this study, we conducted a retrospective chart review of 85 patients undergoing THA. These THA patients were subdivided into three groups based on pre-operative intervention; the first group (SA) did not receive a QL block nor ITM (N=28 control), the second group (SA+QLB) had a QL block only (N=31), and the third group (SA+QLB+ITM) had a QL block and ITM prior to THA. The demographics of the study participants are elaborated in Table 1.

**Table 1:** Demographics of study participants ASA: American Society of Anesthesiologists.

Demographic	Control (n=28)	QL (n=31)	QL+ITM (n=26)
Age (years), mean (SE)	58.96 (2.26)	59.35 (2.65)	59.35 (2.82)
Race/Ethnicity, N (%)			
African American	13 (46%)	17 (55%)	8 (31%)
Native American	0 (0%)	0 (0%)	0 (0%)
Asian	0 (0%)	0 (0%)	0 (0%)
White	15 (54%)	14 (45%)	16 (62%)
Hispanic/Latino	0 (0%)	0 (0%)	1 (4%)
Other	0 (0%)	0 (0%)	1 (4%)
Declined to answer	0 (0%)	0 (0%)	1 (4%)
Sex, N (%)			
Female	13(46%)	18(58%)	13(50%)
Male	15(54%)	13(42%)	13(50%)
BMI, mean (SE)	31.62 (1.07)	29.36 (1.12)	32.43 (1.19)
ASA score, mean (SE)	2.8 (0.08)	2.7 (0.11)	2.7 (0.09)

Our primary outcome measures were the cumulative OME used by patients at 24 and 48 hours and the pain score assessed by the VAS in the PACU and 24 hours postoperative (Table 2). The secondary outcome measures were time to first opioid use, distance ambulated at 24 hours postoperative, and length of hospitalization (Table 2).

**Table 2:** Summary of primary and secondary outcomes PACU: Post-Anesthesia Care Unit; OME: Oral Morphine Equivalents.

Outcome	Control (n=28)	QL (n=31)	QL+ITM (n=26 )	P*
OME, mean (SE)				
24 hours-cumulative	40.4 (5.4)	26.4 (3.5)	19.6 (3.2)	0.0029
48 hours-cumulative	93.1 (13.3)	56.4 (8.5)	53.1 (17.7)	0.044
Pain score, mean (SE), maximum				
PACU	0.64 (0.37), 8	0 (0), 0	0 (0), 0	0.0501
24 hours	3.96 (0.47), 10	1.84 (0.37), 7	1.5 (0.52), 8	0.0004
Time to 1^st^ Opioid Use (hours), mean (SE)	6.59 (1.28)	7.9 (1.29)	12.1 (1.6)	0.0069
Ambulation Distance (feet), mean (SE)				
24 hours	90.8 (13.2)	123.1 (11.4)	103.2 (11.0)	0.117
Length of stay (hours), mean (SE)	45.81 (4.88)	49.23 (4.01)	35.92 (3.75)	0.0058

**Figure 1:**
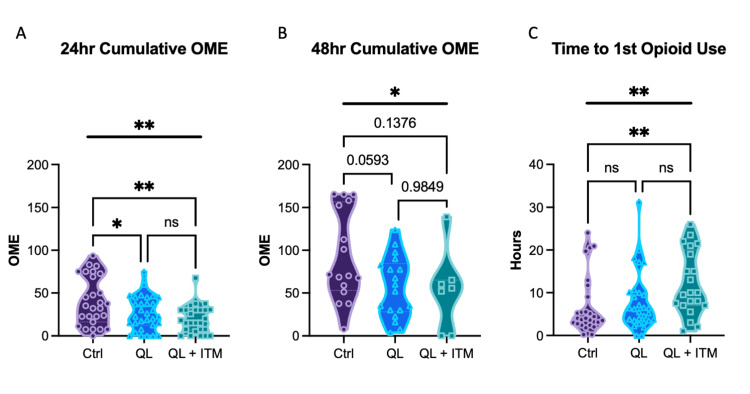
Opioid consumption (1A) 24-hour cumulative oral morphine equivalents (OME) use. (1B) 48-hour cumulative OME use. (1C) Time to first opioid use. One-way ANOVA results are presented above the bolded line; Tukey post hoc comparisons are shown by bracketed lines. Non-significant p-values are denoted with ns or reported numerically. * denotes P≤0.05, ** denotes p≤0.01.

To evaluate the analgesic efficacy of QL and QL plus ITM, we performed an analysis of the electronic Medication Administration Record (MAR) to examine the distribution of pro re nata (PRN) opioid medication over time (Figure 1). OMEs were calculated for patients during their stay at 24 and 48 hours postoperatively. 

Furthermore, the computation of cumulative OMEs within the initial 24 hours postoperatively revealed significant differences. Cumulative OME use at 24 hours was significantly different across the three groups by ANOVA, p=0.0029. Both SA+QLB and SA+QLB+ITM groups had substantially lower cumulative OMEs at 24 hours compared to control (SA) by Tukey post hoc analysis; the mean difference for SA+QLB vs control was 14.05 OME (95% CI of difference 0.306-27.8, p=0.044), and the mean difference for SA+QLB+ITM vs control was 20.8 OME (95% CI of difference 6.454-35.15, p=0.0025). Notably, OME use was not statistically different between the SA+QLB and SA+QLB+ITM groups at 24 hours, with a mean difference of 6.75 OME (95% CI of difference -7.135-20.64, p=0.480) (Figure 1A).

Cumulative OME use at 48 hours was significantly different across the three groups by ANOVA, p=0.044. Both SA+QLB and SA+QLB+ITM groups trended toward lower cumulative OMEs at 48 hours compared to control, but these differences did not meet statistical significance by Tukey post hoc analysis; the mean difference for SA+QLB vs SA was 36.68 OME (95% CI of difference -1.187-74.54, p=0.0593), and the mean difference for SA+QLB+ITM vs SA was 40.02 OME (95% CI of difference -9.916-89.96, p=0.1376). Additionally, OME use was not different at 48 hours between the SA+QLB and SA+QLB+ITM groups, with a mean difference of 3.35 OME (95% CI of difference -45.74-52.43, p=0.9849) (Figure 1B). Time to first use of opioid medication was significantly different across the three groups by Krustal-Wallis, p=0.0069. Dunn’s multiple comparison tests revealed a significantly longer time until first opioid utilization only the SA+QLB+ITM compared to SA, p=0.0052, although there was a trend for a longer time in SA+QLB+ITM compared to SA+QLB alone, p=0.1126 (Figure 1C).

**Figure 2:**
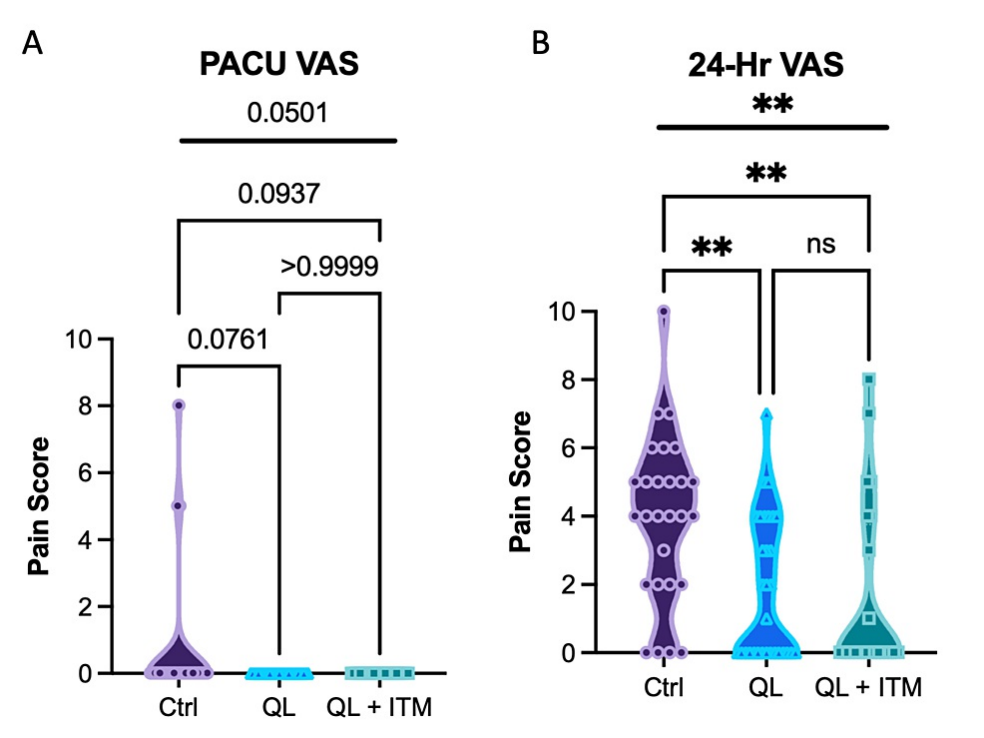
Visual analog scale (2A) Visual analog scale (VAS) for pain assessment in post-anesthesia recovery unit (PACU). (2B) VAS for pain assessment at 24 hours postoperative. One-way ANOVA results are presented above the bolded line; Tukey post hoc comparisons are shown by bracketed lines. Non-significant p-values are denoted with ns or reported numerically. * denotes P≤0.05, ** denotes p≤0.01.

VAS pain scores were assessed in the PACU and at 24 hours postoperatively following THA (Figure 2). Statistical analysis of PACU pain scores was not statistically significantly different between the three groups by one-way ANOVA, p=0.0501. The mean pain scores (95% CI) in the PACU were 0.64 (-0.11-1.40) for SA (Control), 0 (0-0) for SA+QLB, and 0 (0-0) for SA+QLB+ITM (Figure 2A). However, there were statistically significant differences at 24 hours postoperatively, p=0.0004 (Figure 2B); the mean pain score (95% CI) at 24 hours was 3.96 (3.00-4.92) for control, 1.84 (1.08-2.60) for QL, and 1.54 (0.47-2.61) for QL+ITM. Tukey post hoc analysis revealed significantly lower VAS for SA+QLB and SA+QLB+ITM compared to the control, the mean difference 2.12 (95% CI 0.65-3.59, p=0.0025) and 2.42 (95%CI 0.86-3.98, p=0.0012), respectively (Figure 2B). There was no difference between PACU VAS for SA+QLB vs SA+QLB+ITM by post hoc analysis, mean difference of 0.29 (-1.22-1.81, p=0.887) (Figure 2B).

**Figure 3:**
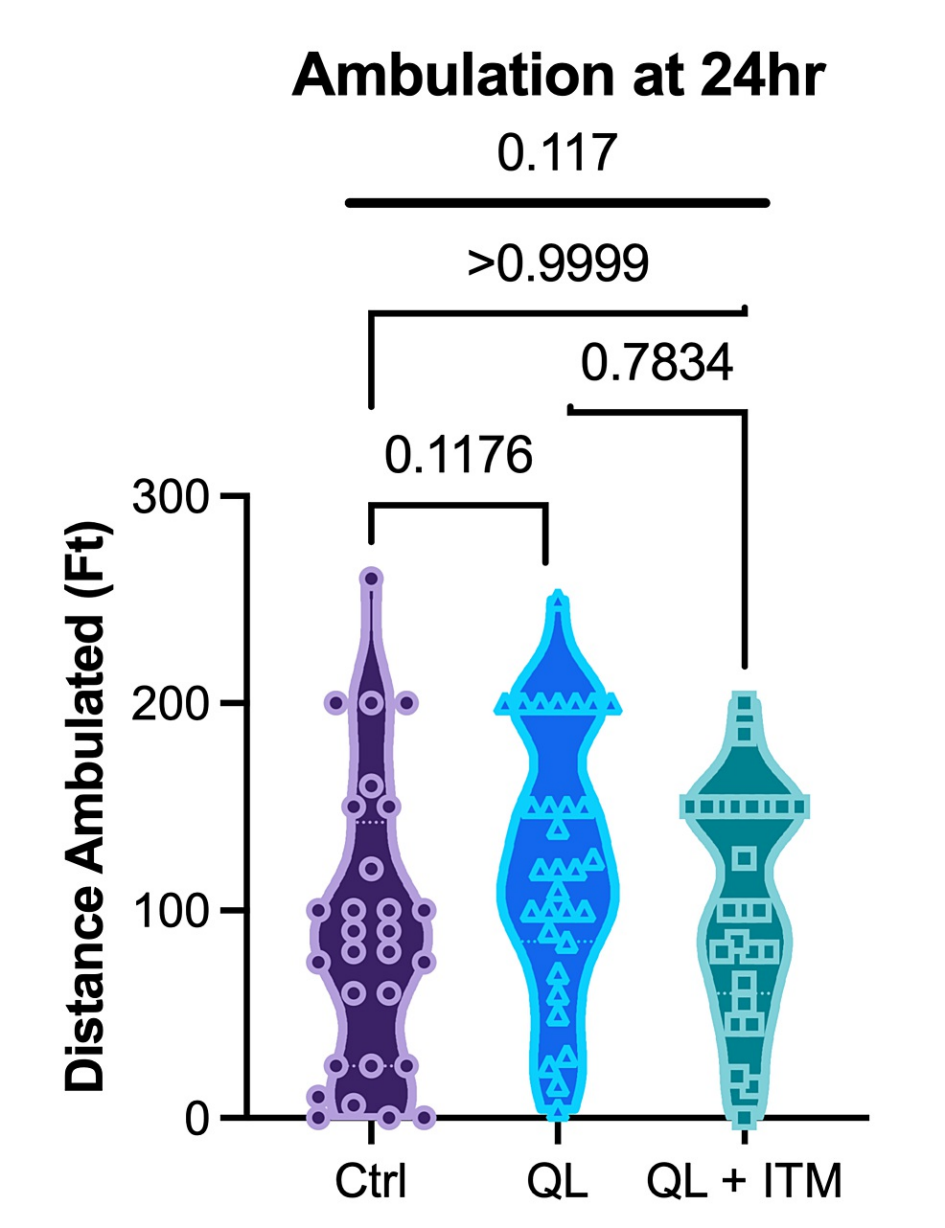
Ambulation distance during physical therapy at 24 hours post operation Ambulation distance during physical therapy at 24 hours post operation. One-way ANOVA results are presented above the bolded line; Tukey post hoc comparisons are shown by bracketed lines. Non-significant p-values are reported numerically.

Ambulation distance during physical therapy at 24 hours postoperation was extracted from the EMR and analyzed as a secondary outcome measure to assess for differences in functional status by group (Figure 3). At the 24-hour mark, the Kruskal-Wallis test did not reveal statistically significant differences in ambulation distance among the three groups (p = 0.117). The mean (SEM) distances were as follows: Control 90.75 feet (13.19), SA+QLB 123.1 feet (11.44), and SA+QLB+ITM 103.2 feet (10.95). There were no significant differences in Dunn's post hoc test comparing control vs. SA+QLB (p=0.1176), control vs. SA+QLB+ITM (p>0.999), or SA+QLB vs SA+QLB+ITM (p = 0.783).

**Figure 4:**
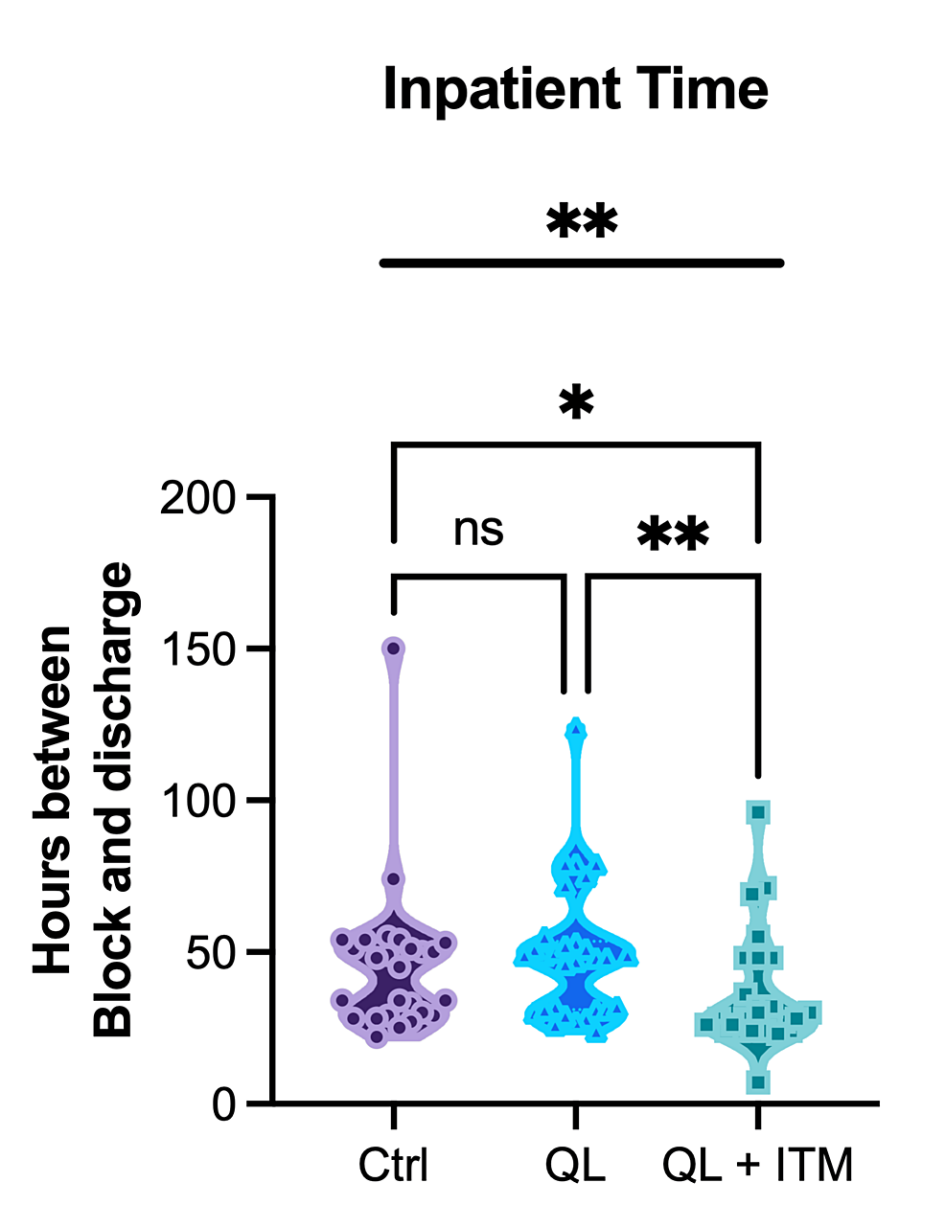
Inpatient time Length of time in hours that the patient remained inpatient, from time of block to discharge. One-way ANOVA results are presented above the bolded line; Tukey post hoc comparisons are shown by bracketed lines. Non-significant p-values are denoted with ns. * denotes P≤0.05, ** denotes p≤0.01.

We assessed whether there was a difference in time spent as an inpatient between control, SA+QLB, or SA+QLB+ITM (Figure 4). The Kruskal-Wallis test indicated a significant difference among the groups (p = 0.0058). The mean (SEM) inpatient times were 45.6 hours (4.88) for control, 49.23 hours (4.01) for SA+QLB, and 35.92 hours (4.8) for SA+QLB+ITM. Dunn's post hoc tests unveiled a significant difference between control and SA+QLB+ITM (p = 0.045) as well as for SA+QLB vs SA+QLB+ITM (p=0.0068). There was no significant difference between control and SA+QLB (p>0.999).

## Discussion

This retrospective study demonstrates that the addition of ITM to QL block provides effective analgesia after primary THA when compared to QL block alone or in the control group. The decision to use a dose of 100 mcg of morphine for our intrathecal injection was based on several studies that suggest this dose provides adequate analgesia with minimal side effects [13].

For our primary efficacy outcome as opioid requirements, our findings suggest that ITM provides a significant decrease in cumulative OME when compared to the control group both at 24 and 48 hours post-surgery. ITM group has lower OME requirements than the QL block-only group but is not statistically significant. The time to first opioid use was significantly delayed in the ITM group, suggesting better analgesia even in the presence of regional block when compared to other groups. We used oral morphine equivalent OME as a more objective measure of analgesia efficacy. The effect was consistent with lower pain scores in the ITM group compared to the control group at earlier time points [14]. The duration of the effect of ITM is estimated to be up to 16 hours [13]. This is consistent with our study results, where we did not find any significant difference in analgesia and OME with the addition of ITM to QL blocks at 24 hours and beyond.

We also demonstrated that there was no difference in PACU VAS scores between the two block groups with and without ITM. This finding can be explained by residual spinal effects during PACU stay. The ITM group has shown increased ambulation distance when compared to the control group. This may be attributed to better pain control in the ITM group with less opioid consumption in the first 24 hours, although not statistically significant when compared to block only group. Also, decreased length of stay in the ITM plus QL group compared with other groups could be related to better analgesia and early ambulation. Inadequately controlled pain after surgery can hinder early mobilization. There are many benefits to the addition of ITM to the multimodal regimen for post-operative analgesia after joint arthroplasty. It has a long duration of action, promotes early mobilization, and decreases hospital stay [15]. Unlike local anesthetics, ITM does not cause muscle weakness, sympathectomy, or loss of proprioception. When compared to parenteral opioids, neuraxial opioids have been found to provide better analgesia and less opioid consumption in certain surgeries [16].

The incidence of PONV, pruritus, respiratory depression, and urinary retention was similar between groups; however, our study was not sufficiently powered to determine whether this finding was statistically significant. Intrathecal morphine has been shown to be associated with an increased risk of PONV, pruritus, and urinary retention, but without any impact on the duration of hospital stay [17]. Although a dose above 100 mcg statistically increased the rate of PONV according to a meta-analysis, we did not find any increased risk of PONV with 100 mcg of ITM when compared to the control. Of note, none of the patients in this retrospective study received dexamethasone, which has been reported to decrease PONV secondary to ITM use from 54% to 22% [18]. We also did not find any incidence of pruritus, urinary retention, or the use of naloxone for respiratory depression.

Because of concerns for sedation, hypoxia, and respiratory depression guidelines for the practice of neuraxial opioids have recommended that patients be continuously monitored for 24 hours after receiving ITM [19]. Respiratory depression has been reported with a high ITM dose of 250 mcg, but recent evidence supports our finding of the absence of respiratory depression with doses at or below 150 mcg [20]. The ITM doses of 100 mcg and 150 mcg provide effective analgesia for patients undergoing lower extremity total knee arthroplasty under spinal anesthesia [21]. In addition, the safety and side effect profile for ITM is similar for both doses as there was no incidence of respiratory depression and antiemetic usage did not differ between all study arms. The ITM dose of 100 mcg did not increase the incidence of sleep apnea or respiratory depression even in older patients undergoing hip arthroplasty [22]. Thus, the safety profile for the use of low-dose ITM (</= 0.15 mg) for analgesia is extremely favorable.

There are some limitations to this study such as small sample size, retrospective design, and publication bias [23]. The study population included patients undergoing THA, and the outcome results may not translate to a different surgical patient population or age group. The sample size in our study is too small to find any statistically significant differences for rare adverse outcomes like respiratory depression and urinary retention. These adverse effects can limit enhanced recovery for same-day arthroplasty, hence future studies with a larger sample size are warranted to confirm this finding. Some results, like ambulation distance, were clinically significant but did not reach statistical significance due to the small sample size as a limitation. The small sample size was intentional, as this study was also a part of the quality improvement initiative. The long-term benefits of ITM could not be assessed due to the retrospective study design.

## Conclusions

There is strong evidence that intrathecal morphine provided effective analgesia after lower extremity arthroplasty. Our study concludes that the addition of 100 mcg ITM for total hip arthroplasty under spinal anesthesia provided improved postoperative analgesia when compared to the control group. Also, the ITM group, when compared to control and block-only groups did better with respect to delay in first opioid use and decreased hospital stay. Our study warrants no more concerns of PONV, pruritus, or respiratory depression with this dose of ITM and requires standard postoperative care. The ideal analgesic dose of ITM for lower extremity arthroplasty under spinal anesthesia is unknown and warrants further investigation. The delineation of the exact role of ITM in the early discharge of THA patients also needs further investigation.
